# The SL–MdDWARF53–MdbHLH1 module regulates *MdAT1*-mediated redox homeostasis and alkaline salt tolerance mechanism in apple

**DOI:** 10.1093/hr/uhag089

**Published:** 2026-03-11

**Authors:** Xiaomin Zhu, Yuqing Zhu, Xiaoyu Zhou, Yong Zhang, Chanyu Wang, Shaoxuan Li, Zhijuan Sun, Qiang Zhao, Xiaodong Zheng, Caihong Wang, Yike Tian

**Affiliations:** College of Horticulture, Qingdao Agricultural University, Qingdao 266109, China; Engineering Laboratory of Genetic Improvement of Horticultural Crops of Shandong Province, College of Horticulture, Qingdao Agricultural University, Qingdao 266109, China; College of Horticulture, Qingdao Agricultural University, Qingdao 266109, China; Engineering Laboratory of Genetic Improvement of Horticultural Crops of Shandong Province, College of Horticulture, Qingdao Agricultural University, Qingdao 266109, China; College of Horticulture, Qingdao Agricultural University, Qingdao 266109, China; Engineering Laboratory of Genetic Improvement of Horticultural Crops of Shandong Province, College of Horticulture, Qingdao Agricultural University, Qingdao 266109, China; Engineering Laboratory of Genetic Improvement of Horticultural Crops of Shandong Province, College of Horticulture, Qingdao Agricultural University, Qingdao 266109, China; State Key Laboratory Breeding Base of Green Pesticide and Agricultural Bioengineering, Key Laboratory of Green Pesticide and Agricultural Bioengineering, Ministry of Education, Guizhou University, Guiyang 550025, China; College of Horticulture, Qingdao Agricultural University, Qingdao 266109, China; Engineering Laboratory of Genetic Improvement of Horticultural Crops of Shandong Province, College of Horticulture, Qingdao Agricultural University, Qingdao 266109, China; Qingdao Academy of Agricultural Sciences, Qingdao 266100, China; Engineering Laboratory of Genetic Improvement of Horticultural Crops of Shandong Province, College of Horticulture, Qingdao Agricultural University, Qingdao 266109, China; College of Life Science, Qingdao Agricultural University, Qingdao 266109, China; College of Horticulture, Qingdao Agricultural University, Qingdao 266109, China; Engineering Laboratory of Genetic Improvement of Horticultural Crops of Shandong Province, College of Horticulture, Qingdao Agricultural University, Qingdao 266109, China; College of Horticulture, Qingdao Agricultural University, Qingdao 266109, China; Engineering Laboratory of Genetic Improvement of Horticultural Crops of Shandong Province, College of Horticulture, Qingdao Agricultural University, Qingdao 266109, China; College of Horticulture, Qingdao Agricultural University, Qingdao 266109, China; Engineering Laboratory of Genetic Improvement of Horticultural Crops of Shandong Province, College of Horticulture, Qingdao Agricultural University, Qingdao 266109, China; College of Horticulture, Qingdao Agricultural University, Qingdao 266109, China; Engineering Laboratory of Genetic Improvement of Horticultural Crops of Shandong Province, College of Horticulture, Qingdao Agricultural University, Qingdao 266109, China

## Abstract

Alkaline salt stress is a key environmental factor restricting the sustainable development of the apple industry, significantly affecting the yield and quality of apple. In recent years, strigolactone (SLs) has been proven to play a central regulatory role in plant stress responses. However, its role and mechanism under alkaline salt stress remain unknown. Based on this, we found that exogenous application of the SL analog GR24^5DS^ can significantly enhance the adaptability of apple to alkaline salt stress. To elucidate the underlying molecular mechanisms, RNA sequencing (RNA-seq) analysis identified the key transcription factor *MdbHLH1*, whose expression was strongly induced by alkaline salt stress. Overexpression of *MdbHLH1* conferred a salt-alkali tolerant phenotype. Further investigation demonstrated that MdbHLH1 directly binds to and activates the promoter of *MdAT1* (Alkali Tolerance 1), a crucial alkali-tolerance gene. The MdbHLH1-*MdAT1* module enhances alkaline salt stress resistance by promoting hydrogen peroxide (H_2_O_2_) efflux and alleviating oxidative damage. More in-depth studies revealed that MdbHLH1 interacts with MdD53 (MdDWARF53), a repressor in the SL signaling pathway. SL signaling induces ubiquitination and degradation of MdD53, thereby releasing MdbHLH1 to activate *MdAT1* expression and ultimately improving alkaline stress tolerance in apple. This study elucidates a key SL–MdD53–MdbHLH1-*MdAT1* regulatory pathway that enhances saline-alkali tolerance in apple by mitigating oxidative stress, thereby providing mechanistic insights into apple’s adaptation to saline-alkali environments.

## Introduction

Apple (*Malus domestica*), as a globally cultivated economic fruit tree, plays an important role in agricultural production [1]. However, soil alkaline salt stress (the synergistic effect of high pH and ion toxicity) is increasingly threatening the growth and development of apple. This leads to a decline in root vitality, a reduction in photosynthetic efficiency, and ultimately a sharp decrease in yield and quality [2, 3]. In the main apple-producing areas in North China and Northwest China, the area of saline–alkaline soil is expanding year by year. Analyzing the molecular mechanism of apple alkali stress tolerance and breeding salt-and-alkali-tolerant varieties have become urgent needs to ensure the sustainable development of the apple industry.

Strigolactones (SLs), a novel class of plant hormones discovered in recent years, are chemically defined as terpenoid lactones derived from carotenoids. It has been well established that SLs play pivotal roles in regulating various plant growth and developmental processes, including but not limited to: suppressing lateral bud outgrowth, promoting adventitious root formation, modulating leaf senescence, and influencing plant height [4, 5]. Moreover, growing evidence underscores the vital role of SLs in helping plants cope with various abiotic stresses, including drought, low temperature, phosphate deficiency, and saline-alkali stress (6–9). For instance, studies have shown that *GmMAX2a* transgenic *Arabidopsis* exhibits enhanced root growth and improved tolerance to both saline-alkali and drought stresses [10]. In alfalfa, Liu *et al.* [11] reported that SL treatment increased soluble sugar and protein content in alfalfa leaves, elevated antioxidant enzyme activities, regulates K^+^/Na^+^ homeostasis, improved photosynthetic performance, and consequently mitigated the detrimental effects of salt stress. However, the specific mechanisms underlying SL-mediated alkaline salt stress tolerance remain largely unexplored, particularly in perennial woody plant species.

In recent years, the core mechanism of SL signal transduction has been clarified. Upon SL perception, the receptor D14 (DWARF14) binds SL and undergoes a conformational change, This change enables D14 to interact with the SCF complex component D3 (DWARF3), forming a D14-D3 ternary complex that recruits the transcriptional repressor D53 (DWARF53). The resulting D14-D3-D53 ternary complex facilitates ubiquitination and degradation of D53, thereby relieving its repression of downstream target genes and ultimately activating the SL signaling pathway [12, 13]. This pathway plays a critical role in plant responses to abiotic stress. For instance, under low-nitrogen conditions, phosphorylation of the rice D14 protein delays its degradation, prolonging SL signal transduction and consequently inhibiting tillering and reducing nutrient consumption [14]. However, whether and how D53, a key transcriptional repressor in the SL signaling pathway, mediates plant responses to alkaline salt stress remains unclear.

D53 plays a pivotal role in the strigolactone signaling pathway by serving as a key node that transmits upstream signals downstream through direct interaction with transcription factors, thereby precisely regulating the gene expression network. For example, in *Arabidopsis*, SMXL6/7/8 proteins interact with transcription factors such as BES1/BZR1 to cooperatively regulate the expression of downstream genes [12, 13]. Among various transcription factor families, the basic helix–loop–helix (bHLH) family has attracted considerable attention due to its large number of members, diverse functions, and critical roles in multiple biological processes. The bHLH transcription factor plays a crucial role in abiotic stress responses [15–17]. For instance, in *Arabidopsis thaliana* bHLH transcription factor AtMYC2, cooperates with the MYB factor AtMYB2 to activate the expression of *RD22*, a key gene in the abscisic acid (ABA) signaling pathway, thereby improving drought tolerance [18]. Under cold stress, the maize bHLH-type transcription factor COOL1 enhances cold tolerance by regulating the expression of the cold-responsive genes *CBF*/*DREB1* and activating pathways associated with membrane stability [19]. Nevertheless, the role of bHLH transcription factors in alkaline salt stress response, and their potential functional interaction with the key repressor D53 within the SL signaling pathway, remain elusive.

Plants subjected to alkaline salt stress experience severe oxidative damage, primarily caused by the excessive accumulation of reactive oxygen species (ROS) such as H_2_O_2_ and O_2_·^−^. Mitigating such oxidative damage relies on efficient ROS scavenging mechanisms within plants. Research indicates that the *AT1* (Alkali Tolerance 1) gene plays a critical role in regulating this process. The γ-subunit of the heterotrimeric G protein encoded by *AT1* modulates H_2_O_2_ efflux, thereby influencing intracellular ROS homeostasis. [20–22]. This regulatory mechanism is highly conserved in gramineous crops such as rice, maize, wheat, and foxtail millet. Notably, the function of this gene exhibits an antagonistic effect between monocots and dicots: specifically, GS3 in rice negatively regulates grain length, while its ortholog AGG3 in *Arabidopsis* positively regulates organ size [23, 24]. However, current research on *AT1* has primarily focused on annual gramineous crops, and its function in perennial woody plants such as apple remains unreported. More importantly, scientific questions such as whether *AT1* is involved in the SL signaling pathway await further investigation.

In summary, this study revealed that SL enhance apple resistance to alkaline salt stress by mitigating oxidative damage. To further elucidate the underlying molecular mechanism, we integrated RNA sequencing (RNA-seq) and chromatin immunoprecipitation sequencing (ChIP-seq) analyses and discovered that the transcription factor MdbHLH1 responds to both SL signaling and alkaline salt stress. This led to the identification of a novel signaling pathway, SL–MdD53–MdbHLH1-*MdAT1*. Our findings not only provide molecular-level insights into the mechanism by which SL regulates the response to alkaline salt stress but also offer a new theoretical perspective for research on alkali tolerance in apple.

## Results

### Strigolactone signaling alleviates growth inhibition and oxidative damage caused by alkaline salt stress

**Figure 1:**
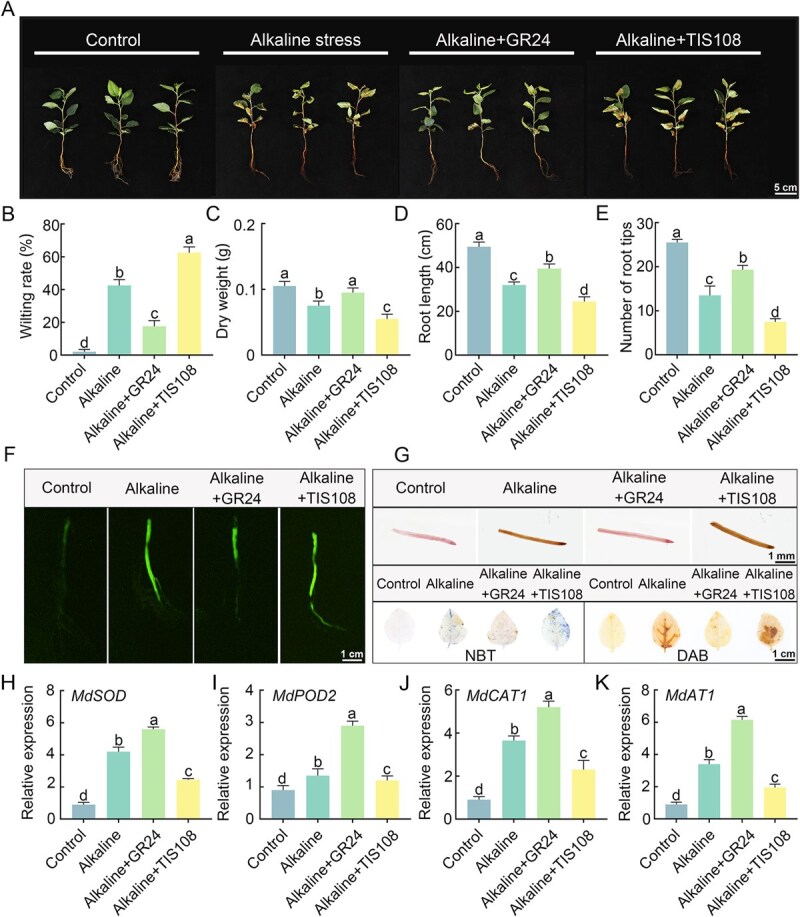
Effects of exogenous GR24^5DS^ and TIS108 on the growth and physiology of *Malus hupehensis* seedlings under alkaline salt stress. (A) Growth phenotypes of *Malus hupehensis* seedlings under control, alkaline salt stress, and treatments with GR24^5DS^ or TIS108; (B) Wilting rate; (C) Dry biomass; (D) Total root length; (E) Root tip number. (F) ROS fluorescence signals in roots; (G) TTC staining of root rhizosphere activity; NBT and DAB staining for leaf ROS detection; (H-K) Relative expression levels of antioxidant enzyme genes under the treatment: (H) *MdSOD*; (I) *MdPOD2*; (J) *MdCAT1*; (K) *MdAT1*. Data represent means ± SD of three biological replicates. Different lowercase letters indicate significant differences according to Fisher’s LSD test (*P* < 0.05). Scale bars: 5 cm (A), 1 cm (F), 1 mm (G).

To investigate the effects of SLs on alkaline salt stress response, *M. hupehensis* seedlings were subjected to 2 days treatments consisting of 50 mM alkaline salt stress, 1 μM exogenous GR24^5DS^ (a strigolactone analog), and 10 μM TIS108 (a strigolactone biosynthesis inhibitor). Compared with the control group, alkaline salt stress significantly inhibited seedling growth (Fig. 1A), as evidenced by a sharp increase in leaf wilting rate (Fig. 1B) and a marked reduction in plant dry weight (Fig. 1C). Application of 1 μM GR24^5DS^ effectively alleviated both the stress-induced wilting and dry weight reduction, whereas 10 μM TIS108 exacerbated these effects. As the primary site of stress perception, the root system exhibited pronounced sensitivity to alkaline salt stress. Our results demonstrated that alkaline salt stress severely suppressed root elongation and development processes, with GR24^5DS^ treatment substantially mitigated these inhibitory effects. Quantitative analysis revealed that while alkaline salt stress reduced total root length to 31.26 cm (44% decrease compared to control), GR24^5DS^-treated plants maintained significantly greater root length (39.73 cm, representing only 27% reduction). TIS108 application exacerbated the stress effects, causing a drastic reduction in root length to 21.76 cm (61% reduction) (Fig. 1D). Alkaline salt stress significantly reduced root tip number, an effect that was partially alleviated by GR24^5DS^ treatment but exacerbated by TIS108 application (Fig. 1E). These results indicate that GR24^5DS^ effectively alleviates alkaline salt stress damage.

Alkaline salt stress induces oxidative damage in plants through excessive accumulation of ROS. Our study demonstrates that GR24^5DS^ effectively alleviates this oxidative damage through multiple protective mechanisms. Under alkaline salt stress, roots exhibited dramatically enhanced ROS production, as shown by intensified fluorescence signals (Fig. 1F) and elevated levels of both H_2_O_2_ and superoxide anion (O_2_·^−^) (Fig. S1B and C), along with significantly increased Malondialdehyde (MDA) content indicating severe membrane lipid peroxidation (Fig. S1A). Histochemical analyses provided visual confirmation of these oxidative effects, with GR24^5DS^ treatment substantially reducing the intensity of nitroblue tetrazolium (NBT) and 3,3′-diaminobenzidine (DAB) staining, while TIS108 treatment produced the opposite effect (Fig. 1G). Notably, 2,3,5-triphenyltetrazolium chloride (TTC) staining revealed that GR24^5DS^ maintained robust root rhizosphere activity despite the stress conditions, consistent with its ability to reduce MDA accumulation (Fig. 1G). At the enzymatic level, GR24^5DS^ treatment significantly enhanced both the expression and activities of key antioxidant enzymes, including superoxide dismutase (SOD), peroxidase (POD), and catalase (CAT). In contrast, TIS108 treatment exhibited the opposite effects, suppressing these antioxidant systems (Fig. 1H–J; Fig. S1D–F). Consistent with this pattern, GR24^5DS^ upregulated the alkaline tolerance gene *AT1*, while TIS108 downregulated its expression (Fig. 1K), confirming the pivotal role of SLs in alkaline stress response. These results establish that SL signaling mediates plant adaptation to alkaline stress through coordinated activation of endogenous antioxidant systems, effectively scavenging ROS and alleviating oxidative damage.

### Transcriptome analysis of the core gene network regulated by SL under alkaline salt stress

**Figure 2:**
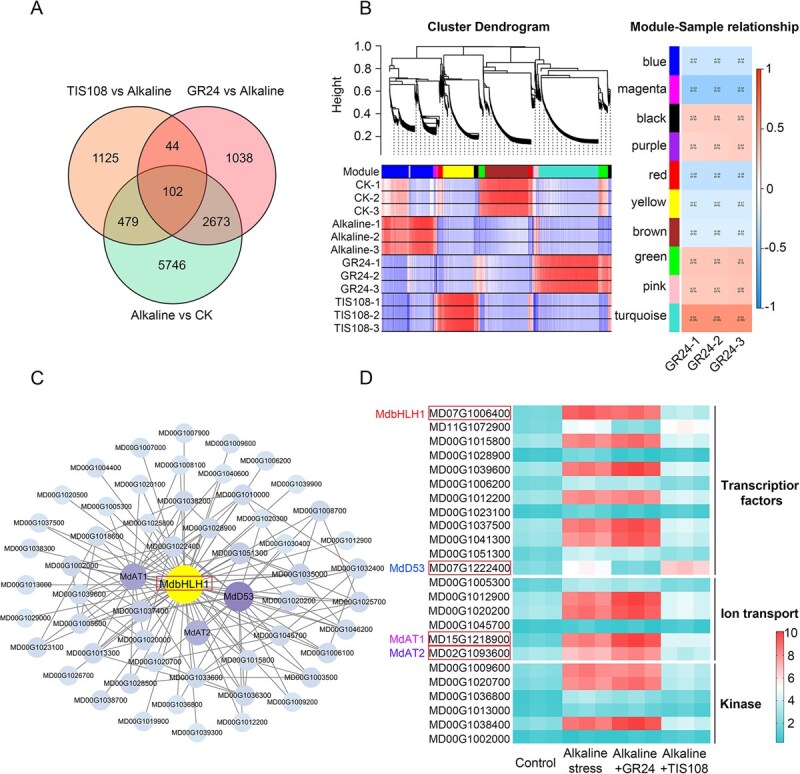
Transcriptome analysis of *Malus hupehensis* plants under alkaline-salt stress and GR24^5DS^ treatment after 6 hours. (A) Venn diagram showing distribution of co-responsive genes under alkaline-salt stress and alkaline-salt stress+GR24^5DS^ treatment; (B) Co-expression clustering dendrogram based on topological overlap of all samples, showing associations between eight gene modules and different treatments. Module-trait correlation values range from −1 to 1, with P-values in parentheses; (C) Cytoscape network interaction diagram of co-expressed genes in brown module; (D) Heatmap of alkaline-salt responsive genes, transcription factors and DEGs in plant hormone signaling pathways.

To analyze the regulatory mechanism of the SL signaling pathway in apple plants under alkaline salt stress, we conducted RNA sequencing (RNA-seq) analysis on *M. hupehensis* seedlings. The experiment included three treatment groups exposed to 50 mM alkaline salt stress, with one group receiving an additional 1 μM GR24^5DS^ treatment, another group receiving 10 μM TIS108 treatment, and all groups being sampled at both 0 and 6 hour time points. Through Venn diagram analysis of differentially expressed genes (DEGs), 102 core genes that responded to all three treatments were identified (Fig. 2A). Gene Ontology (GO) enrichment analysis showed that these DEGs were significantly enriched in categories such as biological processes (e.g. cellular and metabolic processes), cellular components (e.g. membranes and membrane components), and molecular functions (e.g. binding and transport activities) (Fig. S2A). Kyoto Encyclopedia of Genes and Genomes (KEGG) pathway analysis indicated that the DEGs were mainly enriched in pathways such as plant-pathogen interaction and plant hormone signal transduction (Fig. S2B).

Through weighted gene co-expression network analysis (WGCNA), key hub genes in the gene expression network were further identified. The DEGs responsive to alkaline salt and GR24^5DS^ were mainly clustered in the turquoise module, and two core genes, MD07G1006400 and MD15G1218900, were identified (Fig. 2B and C). By integrating the list of alkaline salt-responsive genes, transcription factors, and kinases (Fig. 2D), we determined that the highly expressed *MdbHLH1* (MD07G1006400) was a key transcription factor, and its expression was induced by both alkaline salt stress and GR24^5DS^ treatment. Reverse transcription quantitative PCR verification showed that GR24^5DS^ could significantly up-regulate the expression of *MdbHLH1*. Under alkaline salt stress, *MdbHLH1* expression in GR24^5DS^-treated samples increased 3.8-fold compared to untreated control. In contrast, TIS108 treatment resulted in expression levels that were 2.1-fold lower than control (Fig. S2C). These results further confirmed that *MdbHLH1* was a core gene responsive to alkaline salt and GR24^5DS^, so we selected this gene for in-depth study.

### MdbHLH1 enhances apple alkaline salt tolerance by directly activating the expression of *MdAT1*

**Figure 3:**
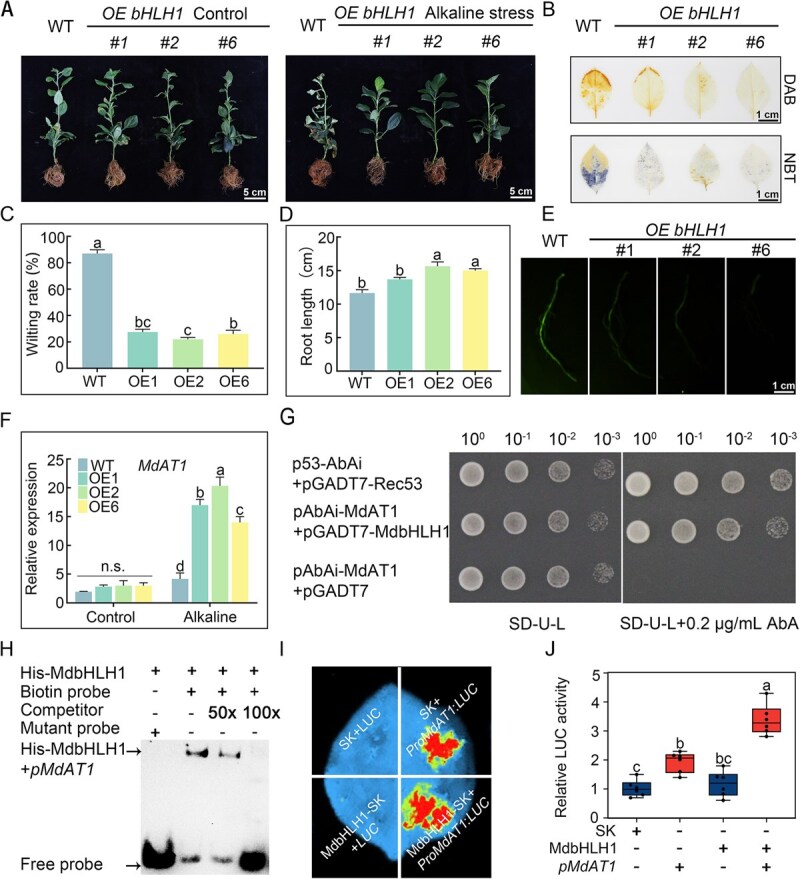
Phenotypic and molecular mechanisms of enhanced alkaline-salt tolerance by *MdbHLH1* overexpression. (A) Phenotypes of WT and OE lines under normal and stress conditions; (B) Trypan blue (cell death) and DAB (H_2_O_2_ accumulation) staining of leaves; (C) Wilting rate under alkaline-salt stress; (D) Root length; (E) ROS fluorescence intensity in roots; (F) Relative expression of *MdAT1* by qRT-PCR; (G) Y1H assay showing MdbHLH1 binding to *MdAT1* promoter; (H) EMSA confirming specific binding of MdbHLH1 to *MdAT1* promoter probe; (I-J) Dual-LUC assay demonstrating MdbHLH1 activation of *MdAT1* promoter. Data represent means ± SD of three biological replicates. Different lowercase letters indicate significant differences (*P* < 0.05) according to Fisher’s LSD test. Scale bars: 5 cm (A), 1 cm (C/D).

To elucidate the functional role of *MdbHLH1* in apple’s response to alkaline salt stress, this study generated three *MdbHLH1*-overexpressing lines (*MdbHLH1-OE* #1, #2, and #6) with expression levels over twice that of wild-type (WT) plants, and two *MdbHLH1*-RNAi transgenic GL3 lines (*MdbHLH1-RNAi* #1, #2) with <50% of WT’s *MdbHLH1* expression (Fig. S3A). Phenotypic observations showed severe wilting and yellowing in WT after stress, with an 89.7% wilting rate. In contrast, *MdbHLH1-OE* lines had significantly improved growth and only 24.3%–34.5% wilting rates (Fig. 3A–C), while RNAi lines exhibited more severe stress symptoms (Fig. S3E). Under stress, RNAi lines’ root length and dry weight were reduced by 40.8 and 37.5% vs. WT (Figs. S3F and G), demonstrating that *MdbHLH1* overexpression alleviates alkaline salt stress.

To investigate the physiological mechanism, histochemical staining was performed. NBT staining showed dark brown coloration (cell death) in WT leaves, which was significantly lighter in *MdbHLH1-OE* lines. DAB staining revealed substantial hydrogen peroxide (H_2_O_2_) accumulation (dark blue patches) in WT, but much less in *MdbHLH1-OE* lines (Fig. 3B), consistent with H_2_O_2_ and O_2_·^−^ quantitative data (Fig. S3B and C). ROS fluorescence intensity in roots was notably stronger in WT than *MdbHLH1-OE* lines (Fig. 3E). Meanwhile, RNAi lines had 54.9 to 59.2% and 50.4 to 59.1% higher levels of these substances vs. WT (Fig. S3H–I), confirming *MdbHLH1* effectively mitigates oxidative damage.

We also examined *AT1* expression in WT and *MdbHLH1-OE* plants. All four genes were significantly up-regulated in *MdbHLH1-OE* lines vs. WT, with *AT1* showing the largest increase (15.9–21.7-fold, Fig. 3F). Given AT1’s known association with alkaline salt stress, we hypothesize it is a key downstream gene in MdbHLH1’s regulatory pathway. Yeast one-hybrid (Y1H) and electrophoretic mobility shift (EMSA) assays confirmed MdbHLH1 directly and specifically binds the *MdAT1* promoter (Fig. 3G and H). Dual-luciferase (Dual-LUC) reporter assays showed a 3.2 LUC/REN ratio in the co-transfected group (Fig. 3I and J), directly demonstrating MdbHLH1 transcriptionally activates the *MdAT1* promoter.

This demonstrates that MdbHLH1 enhances alkaline salt tolerance by directly regulating *MdAT1* to alleviate oxidative damage.

### *MdAT1* confers alkaline salt tolerance by enhancing ROS scavenging

To elucidate the molecular mechanisms and physiological functions of the *MdAT1* gene in plant responses to alkaline salt stress, we generated *MdAT1*-overexpressing transgenic lines (*MdAT1-OE* #1, #2, and #5) and RNA interference lines (*MdAT1-RNAi* #1, #2) (Fig. S4A). In the OE lines, *MdAT1* expression levels were 3–5 times higher than in the WT, whereas in the RNAi lines, expression was reduced to less than half that of the WT.

**Figure 4:**
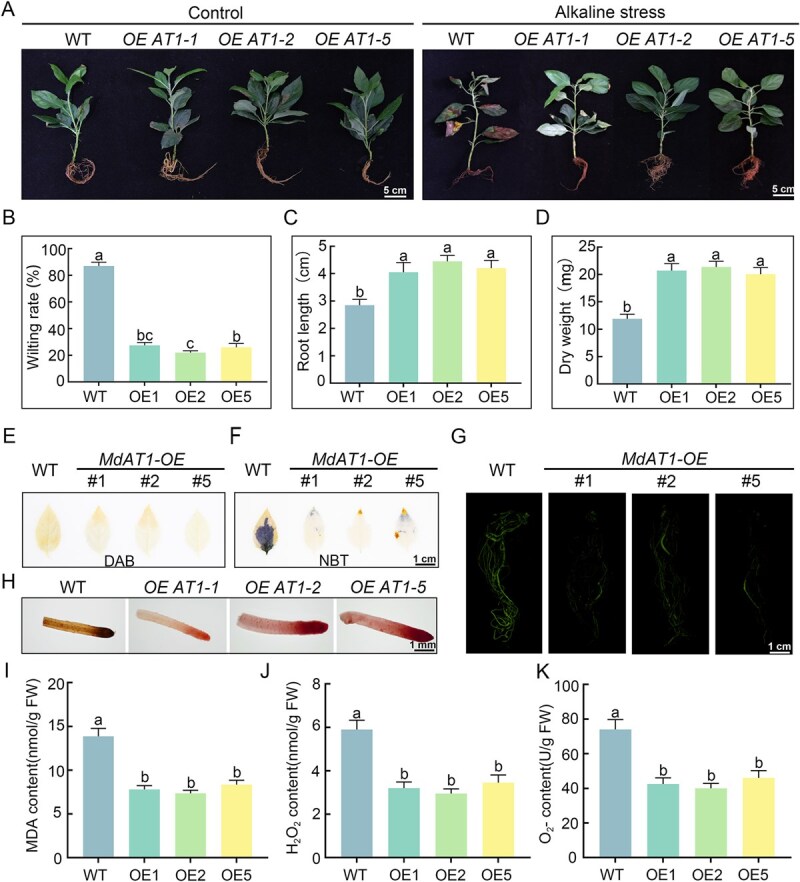
Phenotypic and physiological mechanisms of enhanced alkaline tolerance by *MdAT1* overexpression. (A) Phenotypes of WT and OE lines under control (left) and stress (right) conditions; (B) Wilting rate; (C) Dry biomass; (D) Root length; (E) DAB staining for H_2_O_2_ accumulation; (F) NBT staining for O_2_·^−^ accumulation; (G) ROS fluorescence signals in roots; (H) TTC staining of root rhizosphere activity; (I) Quantitative analysis of MDA content. (J) Quantitative analysis of H_2_O_2_ content; (K) Quantitative analysis of O_2_·^−^ content. Data represent means ± SD of three biological replicates. Different lowercase letters indicate significant differences (*P* < 0.05) according to Fisher’s LSD test. Scale bars: 5 cm (A), 1 cm (E/G),1 mm (H).

Under alkaline salt stress, *MdAT1-OE* lines exhibited significantly enhanced tolerance (wilting rate < 40%) with reduced leaf necrosis, while WT plants showed severe symptoms (wilting rate > 80%) (Fig. 4A). Notably, *MdAT1-RNAi* lines displayed even more severe wilting and necrosis symptoms compared to WT (Fig. S4B). These results indicate that *MdAT1* plays a positive regulatory role in alkaline salt stress tolerance. Consistent with the phenotypic observations, *MdAT1-OE* lines exhibited longer root lengths (average 4.1 ± 0.1 cm) (Fig. 4C) and higher dry weight (nearly twice that of WT) (Fig. 4D). In contrast, *MdAT1-RNAi* lines showed significantly shorter root lengths and reduced dry weight under stress conditions (Fig. S4C and D).

We further analyzed the effect of *MdAT1* on ROS accumulation. NBT and DAB staining revealed significantly reduced accumulation of both O_2_·^−^ and H_2_O_2_ in *MdAT1-OE* lines compared to WT (Fig. 4E and F), which was corroborated by a pronounced reduction in ROS-associated fluorescence signals (Fig. 4G). Quantitative analysis further demonstrated that H_2_O_2_ content in *MdAT1-OE* plants was only 47% of that in WT, accompanied by a 50% reduction in O_2_·^−^ levels and a 42% decrease in malondialdehyde (MDA) content (Fig. 4I–K). In contrast, *MdAT1-RNAi* lines showed significantly elevated levels of both H_2_O_2_ and O_2_·^−^ (Fig. S4E and F). These findings confirm that *MdAT1* likely enhances plant tolerance to alkaline salt stress by effectively regulating ROS scavenging mechanisms and alleviating oxidative damage.

### MdD53 acts as a negative regulator of SL signaling to antagonize the function of MdbHLH1

MdD53, as a key repressor in the SL signaling pathway, plays an important role in plant stress responses. Based on previous research, we successfully screened MdD53 through MdbHLH1 immunoprecipitation mass spectrometry (CoIP-MS). Combining with transcriptome data, we found that the expression of MdD53 was significantly induced by alkaline salt stress (Fig. S2D), suggesting that MdD53 may have an important function in the apple salt-alkali tolerance regulatory network.

**Figure 5:**
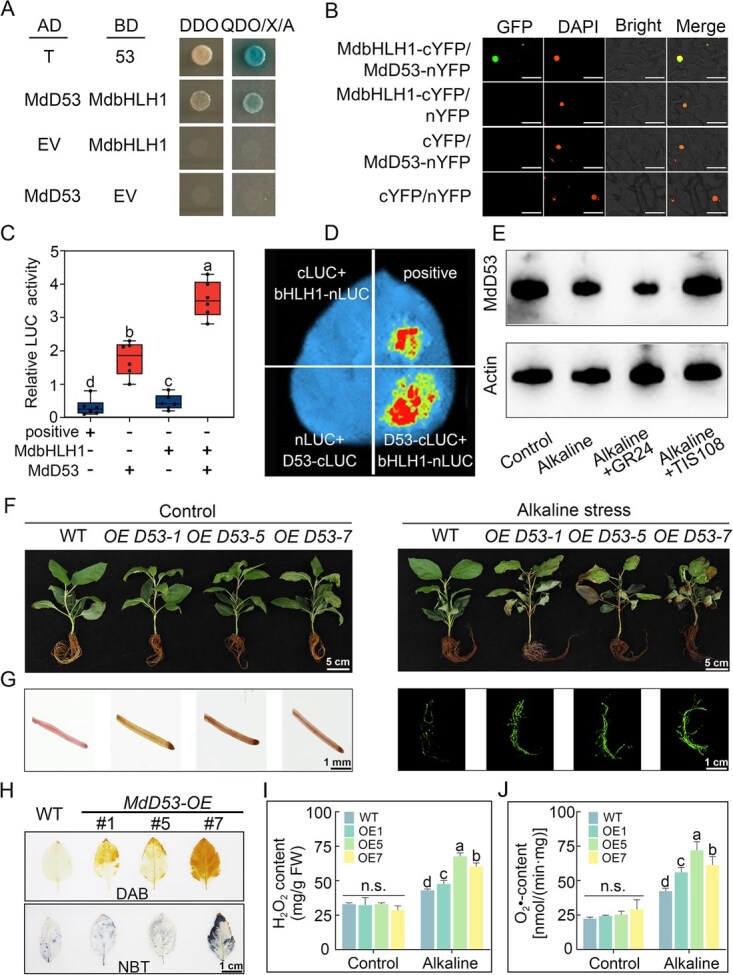
Molecular mechanism of the interaction between MdD53 and MdbHLH1 in regulating apple’s alkaline-salt tolerance. (A) Y2H validation of the interaction between MdD53 and MdbHLH1; (B) BiFC confirmation of MdD53–MdbHLH1 interaction in tobacco epidermal cells; (C) Quantitative measurement of interaction strength by dual-luciferase reporter system; (D) Visualization of protein interaction using luciferase complementation imaging; (E) Western blotting analysis of MdD53 protein accumulation under alkaline-salt stress and SL treatment; (F) Phenotypes of WT and OE lines under normal and stress condition; (G) Detection of ROS fluorescence signals in roots; (H) DAB and NBT staining for leaf ROS detection; (I) Quantitative analysis of H_2_O_2_ content; (J) Quantitative analysis of O_2_·^−^ content. Data represent means ± SD of three biological replicates. Different lowercase letters indicate significant differences (*P* < 0.05) according to Fisher’s LSD test. Scale bars: 5 cm (F), 1 cm (H),1 mm (G).

We first confirmed the interaction between MdD53 and MdbHLH1. Yeast two-hybrid (Y2H) experiments demonstrated that yeast cells co-transformed with MdbHLH1 and MdD53 grew on QDO/X/A medium (Fig. 5A), providing preliminary evidence of protein–protein interaction. Bimolecular fluorescence complementation (BiFC) assays revealed distinct yellow fluorescence signals in tobacco cells (Fig. 5B), visually validating their interaction. Dual-LUC assays showed a 3.2-fold increase in the LUC/REN ratio in co-transfected tobacco leaves (Fig. 5C), and luciferase complementation imaging (Fig. 5D) further confirmed the interaction. Western blot analysis indicated that MdD53 protein levels were significantly elevated under alkaline salt stress. Treatment with the GR24^5DS^ promoted MdD53 degradation, whereas the SL biosynthesis inhibitor TIS108 blocked this degradation (Fig. 5E). These findings suggest that MdD53 interacts with MdbHLH1 and is subject to SL-mediated regulation, thereby implicating it as a potential component of the alkaline salt stress response network.

To further investigate the functional role of MdD53, we generated MdD53-overexpressing apple lines (*MdD53-OE* #1, #5, and #7) and RNA interference lines (*MdD53-RNAi* #1, #2). Under alkaline salt stress, compared with the WT, *MdD53-OE* plants exhibited significantly increased leaf necrosis (Figs 5F and S5A), while *MdD53-RNAi* lines showed markedly alleviated wilting and necrosis symptoms (Fig. S5D and E). This phenotypic observation was consistent with their growth performance: under stress conditions, *MdD53-RNAi* lines had longer root length (25.4% longer than the wild type, Fig. S5F) and higher dry weight (Fig. S5G), whereas *MdD53-OE* lines displayed the opposite trend (Fig. S5B and C). These phenotypes indicate that MdD53 negatively regulates tolerance to alkaline salt stress. In line with this, ROS levels were substantially elevated in *MdD53-OE* lines, as evidenced by significantly stronger ROS-associated fluorescence and pronounced accumulation of O_2_·^−^ and H_2_O_2_ revealed by NBT and DAB staining (Fig. 5H–J). In contrast, the contents of O_2_·^−^ and H_2_O_2_ were significantly reduced in *MdD53-RNAi* lines under stress (Fig. S5H–I).

Taken together, these results indicate that MdD53 interacts with MdbHLH1 and attenuates ROS scavenging capacity, thereby acting as a negative regulator of apple tolerance to alkaline salt stress.

### The MdD53-MdbHLH1-*MdAT1* module cooperatively regulates oxidative stress response

**Figure 6:**
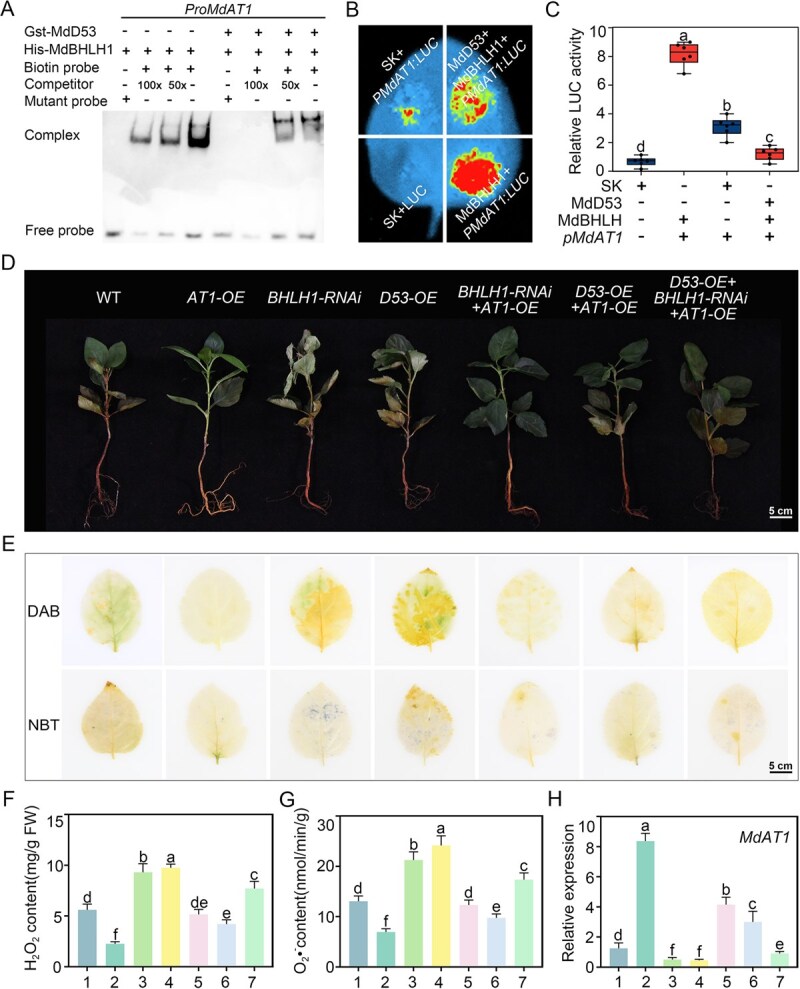
Molecular mechanism of the MdD53–MdbHLH1-*MdAT1* regulatory module in apple alkaline tolerance. (A) EMSA analysis of MdD53’s effect on MdbHLH1 binding to the *MdAT1* promoter; (B-C) Dual-luciferase reporter assay detecting MdD53’s inhibitory effect on MdbHLH1-mediated activation of *MdAT1*; (D) Phenotypic comparison of different genetic materials via transient overexpression under alkaline-salt stress; (E) DAB and NBT staining for ROS detection; (F) Quantitative analysis of H_2_O_2_ content; (G) Quantitative analysis of O_2_·^−^ content; (H) Expression level analysis of *MdAT1* gene. Data represent means ± SD of three biological replicates. Different lowercase letters indicate significant differences (*P* < 0.05) according to Fisher’s LSD test. Scale bars: 5 cm (D/E).

To elucidate the regulatory mechanisms of MdD53, MdbHLH1, and *MdAT1* in apple stress resistance, we performed EMSA assays and found that the addition of MdD53 significantly impaired the binding ability of MdbHLH1 to the *MdAT1* promoter (Fig. 6A). Dual-LUC reporter assays further demonstrated that MdD53 reduced the transcriptional activation of the *MdAT1* promoter by MdbHLH1 by approximately fourfold, indicating that the interaction between MdD53 and MdbHLH1 attenuates the regulatory effect of MdbHLH1 on its downstream target gene *MdAT1* (Fig. 6B and C).

In functional validation, distinct phenotypic differences were observed through the construction of genetic materials (Fig. 6D). *MdAT1*-*OE* plants exhibited enhanced growth, whereas *MdbHLH1*-*RNAi* or *MdD53*-*OE* plants showed growth inhibition. Particularly noteworthy, in the complementary experiment (*MdD53-OE* + *MdAT1-OE*), overexpression of *MdAT1* partially reversed the growth inhibition caused by *MdD53* overexpression, demonstrating that the MdbHLH1-*MdAT1* pathway antagonizes MdD53-mediated growth suppression.

Further exploring the physiological mechanisms, oxidative stress indicators were highly consistent with gene expression data. Quantitative DAB and NBT staining (Fig. 6E) revealed that H_2_O_2_ levels in *MdbHLH1-RNAi* and *MdD53-OE* plants were 2.7-fold and 2.5-fold that of WT, respectively (Fig. 6F), while O_2_·^−^ levels were 2.3-fold and 2.1-fold that of WT (Fig. 6G). In contrast, H_2_O_2_ and O_2_·^−^ contents in *MdAT1-OE* plants were only 62% and 58% of WT levels, and ROS accumulation in the *MdD53-OE* + *MdAT1-OE* line was reduced by 41% compared to the *MdD53-OE* single-transfection line. Subsequent qRT-PCR analysis showed that *MdAT1* expression in *MdAT1-OE* plants was 5.3-fold that of WT, while in *MdbHLH1-RNAi* and *MdD53-OE* plants, it decreased to 31% and 27% of WT levels, respectively (Fig. 6H), with its expression significantly negatively correlated with H_2_O_2_ content.

Based on the above experimental results, this study systematically elucidated the action mechanism of the MdD53–MdbHLH1–*MdAT1* regulatory module from three aspects: molecular interaction, gene function, and physiological response. MdbHLH1 exerts its function by directly activating the *MdAT1* promoter, MdD53 inhibits this process by interacting with MdbHLH1, and *MdAT1*, as a downstream effector, affects the stress resistance of apple by regulating ROS metabolism. These findings provide new theoretical basis for a deeper understanding of the molecular regulatory network of plant stress responses.

## Discussion

Strigolactones (SLs) are an important class of plant hormones, initially known for their roles in regulating branching and promoting mycorrhizal symbiosis [4, 5]. Recent studies have revealed that SLs play a key role in plant responses to abiotic stresses: they enhance drought resistance by modulating stomatal behavior and root architecture [7], and improve cold tolerance through activation of the CBF/DREB1 pathway [25]. In addition, SLs are involved in root remodeling under nutrient stress such as phosphorus deficiency [26]. However, the function of SLs in plant response to alkaline salt stress remains unclear [27], particularly in woody plants, where the molecular mechanisms and involvement of hormone signaling pathways require further investigation. This study systematically elucidates the complete regulatory pathway through which SL enhances alkaline salt tolerance in apple, integrating phenotypic, physiological, and molecular levels of analysis. Phenotypically, we demonstrated that SL treatment significantly improved the resistance of apple plants under alkaline salt stress. Physiologically, SL was found to alleviate oxidative damage. Molecularly, we revealed that SL induces ubiquitination-mediated degradation of the MdD53 protein, thereby releasing its inhibition of the MdbHLH1-*MdAT1* module and forming a comprehensive ‘SL-MdD53–MdbHLH1–*MdAT1*’ signaling pathway. In summary, this study not only identifies *MdAT1* as a major gene responsible for SL-mediated alkaline salt tolerance but also expands and enriches the scientific understanding of SL signal transduction networks.

**Figure 7:**
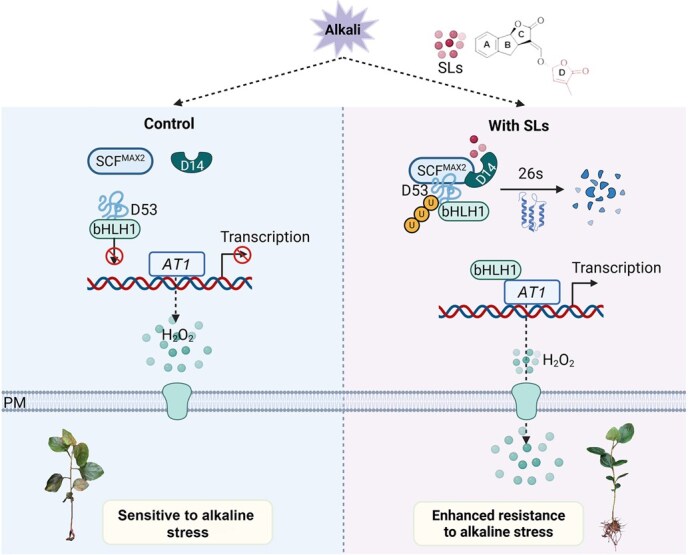
Proposed working model of the SL–MdD53–MdbHLH1–*MdAT1* module in regulating apple alkaline stress tolerance. Left panel (Control/No SL): Under normal conditions or in the absence of SL, the suppressor protein MdD53 interacts with and inhibits the transcription factor MdbHLH1. This prevents MdbHLH1 from binding to the promoter of the *MdAT1* gene, keeping its expression low. Consequently, H_2_O_2_ efflux is reduced, and the plant remains sensitive to alkaline stress. Right panel (With SL): Upon SL perception, the SL receptor (MdD14) and the SCFMAX2 ubiquitin ligase complex facilitate the ubiquitination and subsequent degradation of MdD53. The degradation of MdD53 releases MdbHLH1 from inhibition. The active MdbHLH1 then activates the transcription of *MdAT1*, which encodes a protein that promotes H_2_O_2_ efflux. The enhanced H_2_O_2_ scavenging capacity improves oxidative stress homeostasis, ultimately leading to enhanced alkaline stress resistance in apple.

In recent years, research on D53/SMXL proteins has extended beyond their classical role in regulating shoot branching, increasingly revealing the crucial functions of these core repressor proteins in the SL signaling pathway during abiotic stress responses [12]. Studies have shown that in *Arabidopsis smxl6/7/8* mutants, enhanced expression of SnRK2.3 improves drought resistance and increases sensitivity to ABA [28]. D53 interacts with the transcription factor GRF4 to suppress the expression of nitrogen metabolism-related genes, thereby affecting nitrogen uptake and utilization [29]. These findings suggest that D53 family proteins may act as hub nodes integrating SL signaling with multiple stress responses. However, whether D53 is involved in regulating alkaline salt stress-particularly its interaction mechanism with transcription factors related to salt-alkali tolerance-remains a research gap in woody plants. This study for the first time reveals the interaction mechanism between MdD53 and the alkaline salt-responsive transcription factor MdbHLH1 in apple. Through experiments such as IP-MS, Y2H, and BiFC, we confirmed that MdD53 interacts with MdbHLH1 to inhibit its transcriptional activation function on the downstream target gene *MdAT1* (Fig. 5). This inhibitory effect can be relieved by the degradation of MdD53 induced by SL, thus discovering a new ‘SL-MdD53-MdbHLH1-*MdAT1*’ signaling pathway in apple (Fig. 7). This discovery not only expands our understanding of the functions of D53 proteins but also provides a new perspective for the study of the SL signal transduction network in woody plants.

In plant responses to alkaline salt stress, the precise regulation of H_2_O_2_ metabolism is critically important. Studies have shown that the classic alkali tolerance gene *AT1* and its homologs exhibit significant functional divergence across species. In gramineous crops, AT1/GS3 encodes an atypical G-protein γ subunit that enhances alkali tolerance by regulating aquaporin activity and promoting H_2_O_2_ efflux [21]. Notably, this gene exhibits antagonistic effects in monocots and dicots: rice GS3 negatively regulates grain length, while its *Arabidopsis* ortholog AGG3 positively regulates organ size [23, 24]. This phenomenon of ‘conserved molecular function but opposing phenotypic effects’ suggests that AT1/GS3/AGG3 may mediate distinct signaling networks in different plant lineages. Against this background, this study for the first time identified and functionally verified the homologous gene of *AT1*, *MdAT1*, in apple. We found that alkaline salt stress significantly induced the expression of *MdAT1*, and its over-expressing plants showed higher survival rates and biomass accumulation (Fig. 4). Mechanistically, we confirmed that *MdAT1* significantly reduced the ROS level accumulated under stress by promoting the efflux of H_2_O_2_, thereby alleviating oxidative damage (Fig. 4). This function indicates that in apple, *MdAT1* inherits the core molecular function of alkali-stress tolerance, but its potential role in growth and development remains to be further explored.

In summary, this study systematically elucidated the molecular mechanism by which the SL signaling pathway regulates the tolerance of apple to alkaline salts. Our research findings indicate that in the absence of exogenous SL, MdD53 interacts with MdbHLH1 to form a protein complex, inhibiting the binding and transcriptional activation ability of MdbHLH1 to the promoter of the downstream target gene *MdAT1*. However, in the presence of SL, MdD53 undergoes ubiquitination and degradation, thus relieving the inhibition of MdbHLH1. The released MdbHLH1 subsequently activates the expression of *MdAT1*, effectively reduces the intracellular ROS level by promoting the efflux of H_2_O_2_, alleviating oxidative damage, and ultimately significantly enhancing the tolerance of apple to alkaline salt stress (Fig. 7). It should be emphasized that the SL-MdD53-MdbHLH1-*MdAT1* regulatory module revealed in this study is just one important signaling pathway by which apple respond to alkaline salt stress that we have discovered. Future research will continue to delve into whether the SL signal also regulates the ion homeostasis process through other downstream components, as well as the crosstalk mechanism between this pathway and other known saline-alkali tolerance signaling pathways. This will further improve the theoretical framework of the apple saline-alkali tolerance regulatory network, providing new molecular targets and theoretical basis for apple stress-resistant breeding.

## Materials and methods

### Plant materials

Tissue-cultured *M. hupehensis* and Gala3 (GL3) apple plants were subcultured every 21 days on a subculture medium [4.43 g·L^−1^ Murashige and Skoog (MS) medium +0.5 mg·L^−1^ indole-3-butyric acid (IBA) + 0.5 mg·L^−1^ 6-benzylaminopurine (6-BA)]. Thirty days after subculture, the apple plants were transferred to a rooting medium (2.215 g·L^−1^ MS medium +0.5 mg·L^−1^ IBA, pH = 4.5). After 30 days of rooting, they were transplanted into soil for acclimatization treatment for 15 days, and subsequently used for alkaline salt stress and SL treatment. All apple plants were cultured in an artificial climate chamber with an ambient temperature of 24 ± 1°C, a light/dark cycle of 16/8 hours, and a light intensity of 150 μmol·m^−2^·sec^−1^.

### Alkaline salt stress and GR24^5DS^ treatment

Thirty days after cultivation on rooting medium, 120 tissue-cultured plantlets of *M. hupehensis* with similar growth status were selected and randomly divided into four groups, with 30 plants per group. The plantlets were then transplanted into nutrient soil and acclimated for fifteen days before all seedlings were transferred to a hydroponic system for treatment. After a 48-hour treatment period, phenotypic observations and photography were conducted. The four treatment groups were set as follows: group I apple plants were cultivated with water irrigation as the control; group II was cultivated with 50 mM alkaline salt (sodium carbonate: sodium bicarbonate = 1:1, pH = 8.5) irrigation; group III was cultivated with irrigation of 50 mM alkaline salt and 1 μM GR24^5DS^; group IV was cultivated with irrigation of 50 mM alkaline salt and 10 μM TIS108. GR24^5DS^ and TIS108 were dissolved in dimethyl sulfoxide (DMSO) and stored at −20°C.

Stable transgenic apple plants (*MdbHLH1*-OE, *MdAT1*-OE, and *MdD53*-OE; *MdbHLH1*-RNAi, *MdAT1*-RNAi, and *MdD53*-RNAi) were acclimatized using the same hydroponic method (Figs 3–5, Figs S3–S5). A total of 60 tissue-cultured plantlets each of wild-type and transgenic ‘Gala’ apple plants with similar growth status were selected and randomly divided into 2 groups, with 30 plantlets per genotype in each group. All plants were maintained in a hydroponic system throughout the experiment. Group I (Control) was cultivated with water, while Group II was cultivated with a 50 mM alkaline salt solution (sodium carbonate: sodium bicarbonate = 1:1, pH 8.5). The volume of the hydroponic solution was 5 liters. Samples were collected 6 hours after the start of treatment, flash-frozen in liquid nitrogen, and stored at −80°C for subsequent RNA and protein extraction. Phenotypic data-including wilting rate, fresh weight, dry weight, and root-related parameters-were recorded two days after treatment. The experimental concentrations and treatment procedures were performed in accordance with the methods described in Umehara *et al.* [5], Ito *et al.* [30], and Zhang *et al.* [31].

The *MdD53-pBI121*, *MdbHLH1-pBI121*, *MdAT1-pBI121, anti-MdD53-pBI121*, *anti-MdbHLH1-pBI121*, and *anti-MdAT1-pBI121* constructs were transiently transformed into the seedlings of *M. hupehensis* via agroinfiltration (Fig. 6). After agroinfiltration, plants were kept in darkness for 24 hours, followed by cultivation under light for 48 hours (when transient expression was expected to be at a high level), the seedlings were then subjected to alkaline salt stress treatment by cultivation in a 50 mM solution under hydroponic conditions. Tissue samples for RNA and protein analysis were collected 6 hours after the onset of stress treatment, flash-frozen in liquid nitrogen, and stored at −80°C. Phenotypic data (wilting rate, fresh weight, etc.) of the plants were recorded 2 days after the stress treatment.

### Determination of oxidative damage index and antioxidant enzyme activity

ROS staining of roots was conducted as described by Li *et al.* [32]. Clean roots were immersed in 5-(and 6)-chloromethyl-2′,7′-dichlorodihydrofluorescein diacetate (CM-H_2_DCFDA) solution and subjected to a vacuum for 30 min. Excess CM-H_2_DCFDA was then washed away using distilled water. Root samples were observed and photographed under a plant in vitro fluorescence detector (Vilber Bio Imaging, Paris, France). The malondialdehyde (MDA) content of roots was measured as described by Zheng *et al.* [33].

Activities of superoxide dismutase (SOD), peroxidase (POD), and catalase (CAT) were measured as described by Li *et al.* [34] using 0.1 g of roots.

### Library construction, transcriptome sequencing (RNA-seq), and data analysis

Roots and leaves of *M. hupehensis* tissue culture plants under control, alkaline salt stress, alkaline salt + GR24^5DS^, and alkaline salt + TIS108 treatments were selected for RNA-seq analysis, with three independent biological replicates for each tissue type. Total RNA was extracted from samples and purified using an RNA prep Pure Plant Total RNA Extraction Kit (Qiagen, Japan). RNA samples (1 μg RNA per sample) were sent to Biomarker Technologies Corporation (Beijing, China) for sequencing on an Illumina HiSeq 2000 system using the single-end mode. Library preparation, clustering and sequencing, quality control, reads mapping, and gene ontology (GO), and Kyoto Encyclopedia of Genes and Genomes (KEGG) enrichment analyses of DEGs were conducted as described by Li *et al.* [35]. The reference genome was GDDH13v1.1 (https://www.rosaceae.org/species/malus/malus).

### Reverse-transcription quantitative polymerase chain reaction

RNA was extracted according to the instructions of a rapid RNA extraction kit (Tiangen, Beijing, China). Reverse transcription of cDNA was performed using a SPARKscript II RT Plus Kit (SparkJade, Jinan, China), and reverse-transcription quantitative polymerase chain reaction (RT-qPCR) assays were conducted as described by Zheng *et al.* [36] using ChamQ™ SYBR® qPCR Master Mix to detect the relative expression levels of genes. The RT-qPCR protocol included annealing at 94°C for 3 min, followed by 44 cycles of 94°C for 10 sec and 60°C for 30 sec. Relative levels were determined using the 2^^-ΔΔ*Ct*^ method. Four biological replicates were analyzed for each sample. MdActin (MDP0000774288) was used as an internal control. Primers were designed using Primer 6 (http://www.premierbiosoft.com/primerdesign/) software and checked through a BLAST search of the apple genomic database. Primer sequences are shown in Table S1.

### Generation of transgenic apple plants

The coding sequences (CDSs) of *MdD53* (MD07G1222400), *MdbHLH1* (MD07G1006400), and *MdAT1* (MD15G1218900) were amplified from ‘GL-3’ using the primers shown in Supplemental DataSet 1. Each CDS was then individually cloned into the *pBI121* vector to generate the following overexpression constructs: *35S: MdD53-pBI121* (*OE MdD53*), *35S: MdbHLH1-pBI121* (*OE MdbHLH1*), and *35S: MdAT1-pBI121* (*OE MdAT1*). Meanwhile, the antisense sequences *anti-MdD53-pBI121*, *anti-MdbHLH1-pBI121*, and *anti-MdAT1-pBI121* were cloned into the *pBI121* vector to generate the RNAi interference constructs *RNAi MdD53*, *RNAi MdbHLH1*, and *RNAi MdAT*1. The recombinant vectors were introduced into *Agrobacterium tumefaciens* strain EHA105 and used to transform ‘GL-3’ plants following a previously described method [37]. Transgenic apple plants were verified by PCR and RT-qPCR analyses.

### EMSA assay

The coding sequence (CDS) of *MdbHLH1* was cloned into the pET-32a vector (HIS tag). The HIS–MdbHLH1 fusion protein was expressed in *Escherichia coli* (BL21) and purified as previously described [36]. For electrophoretic mobility shift assay (EMSA), promoter fragments of *MdAT1* containing the E-box element were used as biotin-labeled oligonucleotide probes or unlabeled competitor oligonucleotides (Thermo Fisher Scientific). EMSA was performed using a Light Shift Chemiluminescent EMSA Kit (Thermo Fisher Scientific), with HIS protein serving as a negative control.

### Dual-LUC assay

The promoter of *MdAT1* was cloned into the pGreenII 0800 LUC vector upstream of the LUC gene as the reporter vector. The CDSs of *MdD53/MdbHLH1* were digested with XbaI/SmaI and cloned into the pGreenII 62-SK vector as effector vectors, with the primers used listed in Table S1. Combinations of reporter and effector vectors (*ProMdAT1:LUC* + SK, *ProMdAT1:LUC* + MdbHLH1-SK, *ProMdAT1:LUC* + MdD53-SK + MdbHLH1-SK) carried by *A. tumefaciens* GV3101 were transiently co-expressed in the leaves of one-month-old *Nicotiana benthamiana*. With reference to the method described by Zheng *et al.* [38], LUC activity was detected 3 days after transformation using a Living Plant Fluorescence Detector (Vilber, Paris, France). The ratio of LUC to Renilla luciferase (REN) activity (Novogene, Beijing, China) was used to quantify the relative LUC activity.

### Bimolecular fluorescence complementation assays

To verify the interaction between MdD53 and MdbHLH1, the CDS of *MdD53* was inserted into the nYFP vector, and the CDS of *MdbHLH1* was inserted into the cYFP vector, which were then introduced into *A. tumefaciens* strain GV3101, respectively. Onion (*Allium cepa*) epidermis was dark-cultured on 1/2 MS medium for 1 day (ambient temperature 24 ± 1°C), and then infiltrated with GV3101 bacterial solutions carrying the following combinations: MdD53-nYFP + MdbHLH1-cYFP, MdD53-nYFP + cYFP, nYFP + MdbHLH1-cYFP, and cYFP + nYFP. Two days later, green fluorescence was observed using an Olympus confocal microscope (Thermo Fisher Scientific).

### Yeast two-hybrid assay

The CDS of *MdD53* was inserted into the pGADT7 vector digested with EcoRI/SmaI to construct MdD53-AD, and the CDS of *MdbHLH1* was inserted into the pGBKT7 vector digested with SmaI/SalI to construct MdbHLH1-BD. The primers used are listed in Table S1. Subsequently, the MdD53-AD and MdbHLH1-BD plasmids were co-transformed into Saccharomyces cerevisiae strain Y2H Gold. Meanwhile, HB2-AD + HB2-BD (positive control), MdD53-AD + BD (negative control), and AD + MdbHLH1-BD (negative control) were transformed as controls. Yeast cells grown on SD-Leu/-Trp medium were used as transformation controls, and colonies were transferred to SD/-Leu/-Trp/-His/-Ade medium containing X-α-Gal (SolarBio, Beijing, China) for interaction detection. The Y2H assay was performed according to the method described by Zhou *et al.* [39].

### Statistical analysis

Data were subjected to analysis of variance (ANOVA) followed by Fisher's least significant difference (LSD) or Student’s *t*-test analyses. Statistically significant differences at *P* < 0.05 are indicated. Statistical computations were carried out using SPSS software (IBM, Armonk, NY, USA).

### Accession numbers

The RNA-seq data has been deposited in the National Center for Biotechnology Information Sequence Read Archive under associate code PRJNA1330522.

Sequence data from this article can be found in the GenBank/EMBL data libraries under accession numbers: *MdDWARF53* (*MD07G1222400*), *MdbHLH1* (*MD07G1006400*), and *MdAT1* (*MD15G1218900*)*.*

## Supplementary Material

Web_Material_uhag089

## Data Availability

The data supporting the findings of this study are available within the paper figures and the Supplementary Information.
