# Supplementary material for: The SL–MdDWARF53–MdbHLH1 module regulates MdAT1-mediated redox homeostasis and alkaline salt tolerance mechanism in apple
Source: Hortic Res. 2026 Mar 11;13(7):uhag089. doi: 10.1093/hr/uhag089 (PMC13271796; doi:10.1093/hr/uhag089)
Supplement: Web_Material_uhag089 [file web_material_uhag089.zip › SUPPORTING INFORMATION.docx]

**SUPPORTING INFORMATION**

The following materials are available in the online version of this article.


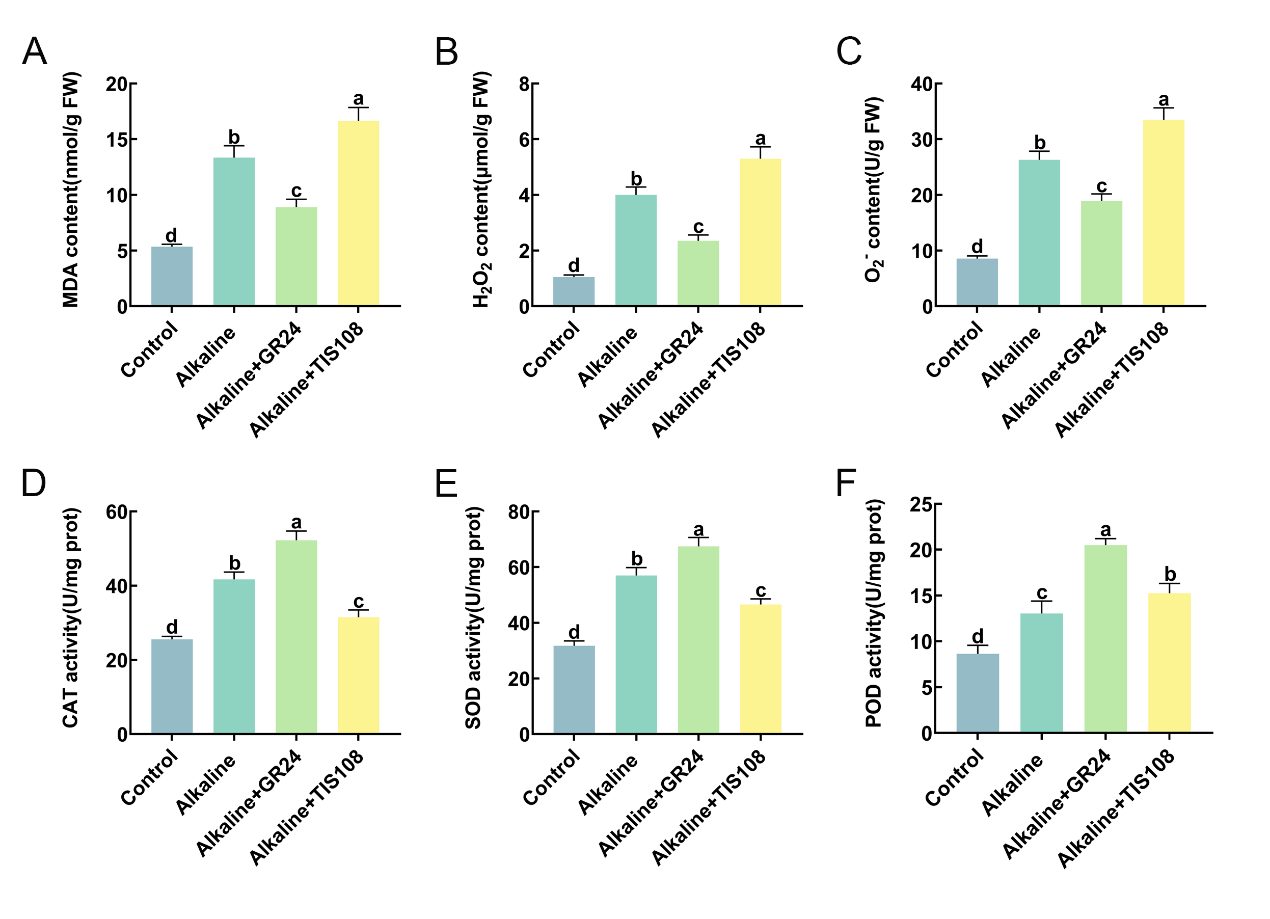


**Figure S1.** Effects of exogenous GR24^5DS^ and TIS108 on the growth and physiological characteristics of *Malus hupehensis* seedlings under alkaline salt stress. (A) MDA content; (B) H_2_O_2_ content; (C) O_2_·^-^ content; (D-F) Antioxidant enzyme activities under different treatments: (D) CAT; (E) SOD; (F) POD. Data represent means ± SD of three biological replicates. Different lowercase letters indicate significant differences according to Fisher’s LSD test (*P*<0.05).


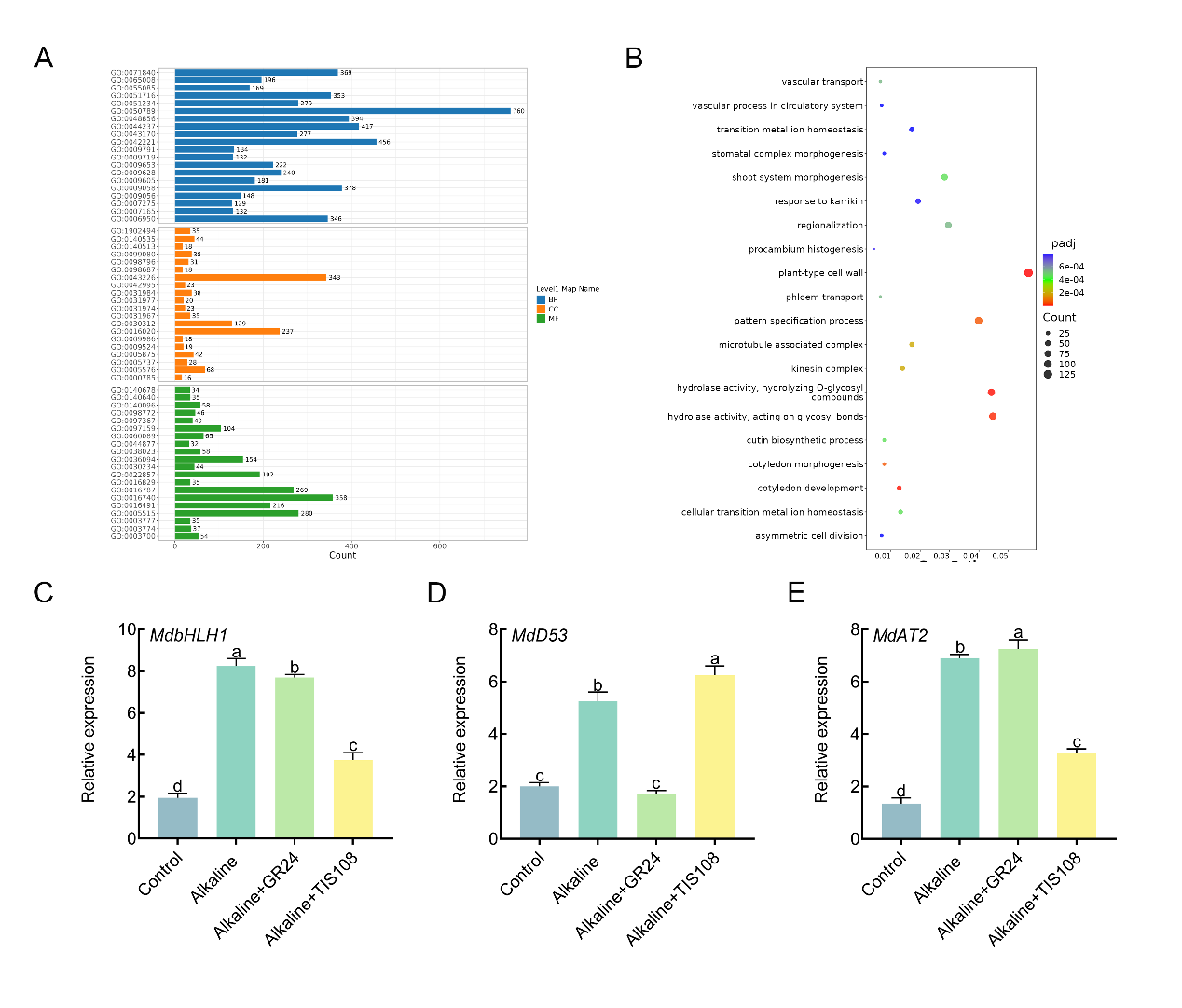


**Figure S2.** GO and KEGG pathway enrichment analyses of differentially expressed genes (DEGs) and expression patterns of related genes in *Malus hupehensis* under alkaline salt stress with exogenous GR24^5DS^ treatment. (A) GO enrichment analysis; (B) Bubble chart of KEGG pathway enrichment analysis of DEGs from RNA-seq data of *Malus hupehensis* treated with alkaline salt stress and exogenous GR24^5DS^ for 6 hours; (C-E) Relative expression levels of *MdbHLH1* (C), *MdD53* (D), and *MdAT2* (E) under different treatments. Different lowercase letters indicate significant differences according to Fisher’s LSD test (*P*<0.05).


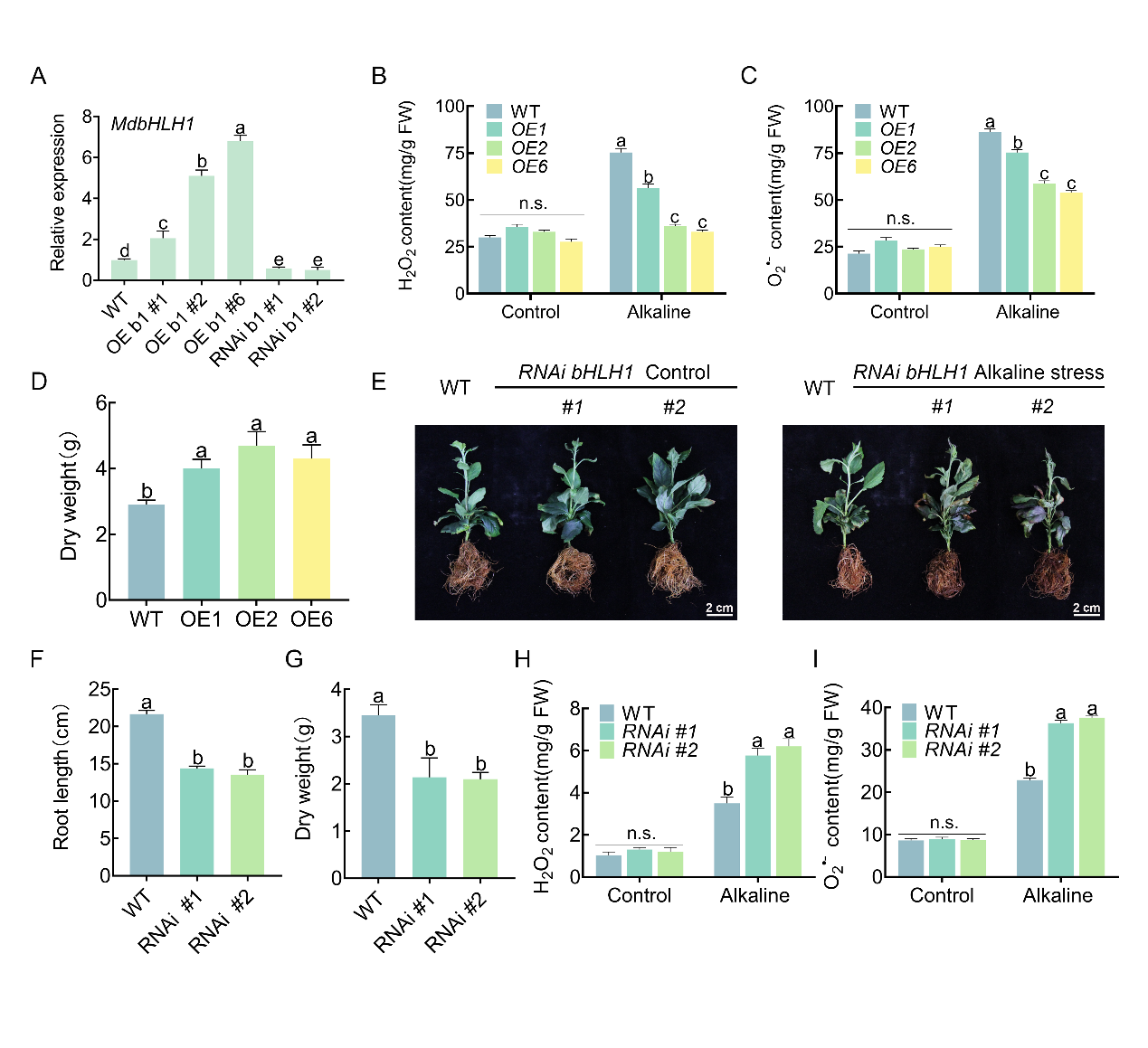


**Figure S3.** Identification of *MdbHLH1*-overexpressing (OE) and RNA interference (RNAi) transgenic apple plants (A); Physiological indices of each line under alkaline salt stress: Contents of H_2_O_2_ (B) and O_2_·^-^ (C), as well as dry weight (D) in *MdbHLH1-OE* lines and wild-type (WT); Phenotype (E), Root length (F), Dry weight (G), contents of H_2_O_2_ (H) and O_2_·^-^ (I) of *MdbHLH1-RNAi* lines under alkaline salt stress. Data represent means ± SD of three biological replicates. Different lowercase letters indicate significant differences according to Fisher’s LSD test (*P*<0.05). Scale bars: 2 cm (E).


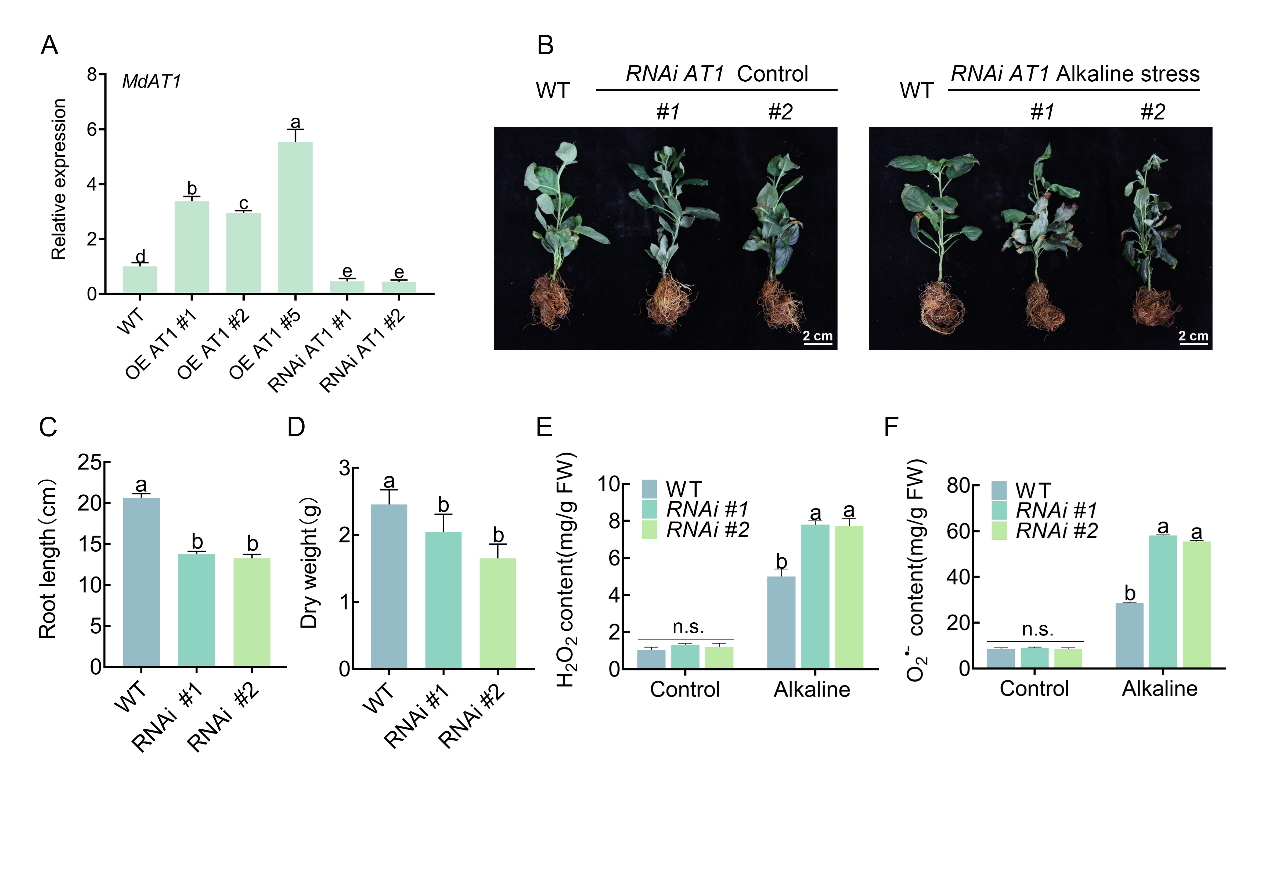


**Figure S4.** Identification of *MdAT1*-overexpressing (OE) and RNA interference (RNAi) transgenic apple plants (A); Phenotypic and physiological indices of *MdAT1-RNAi* lines under alkaline salt stress: Alkaline salt stress phenotype of *MdAT1-RNAi* lines (B); Root length (C); Dry weight (D); Contents of H_2_O_2_ (E) and O_2_·^-^ (F). Data represent means ± SD of three biological replicates. Different lowercase letters indicate significant differences according to Fisher’s LSD test (*P*<0.05). Scale bars: 2 cm (B).


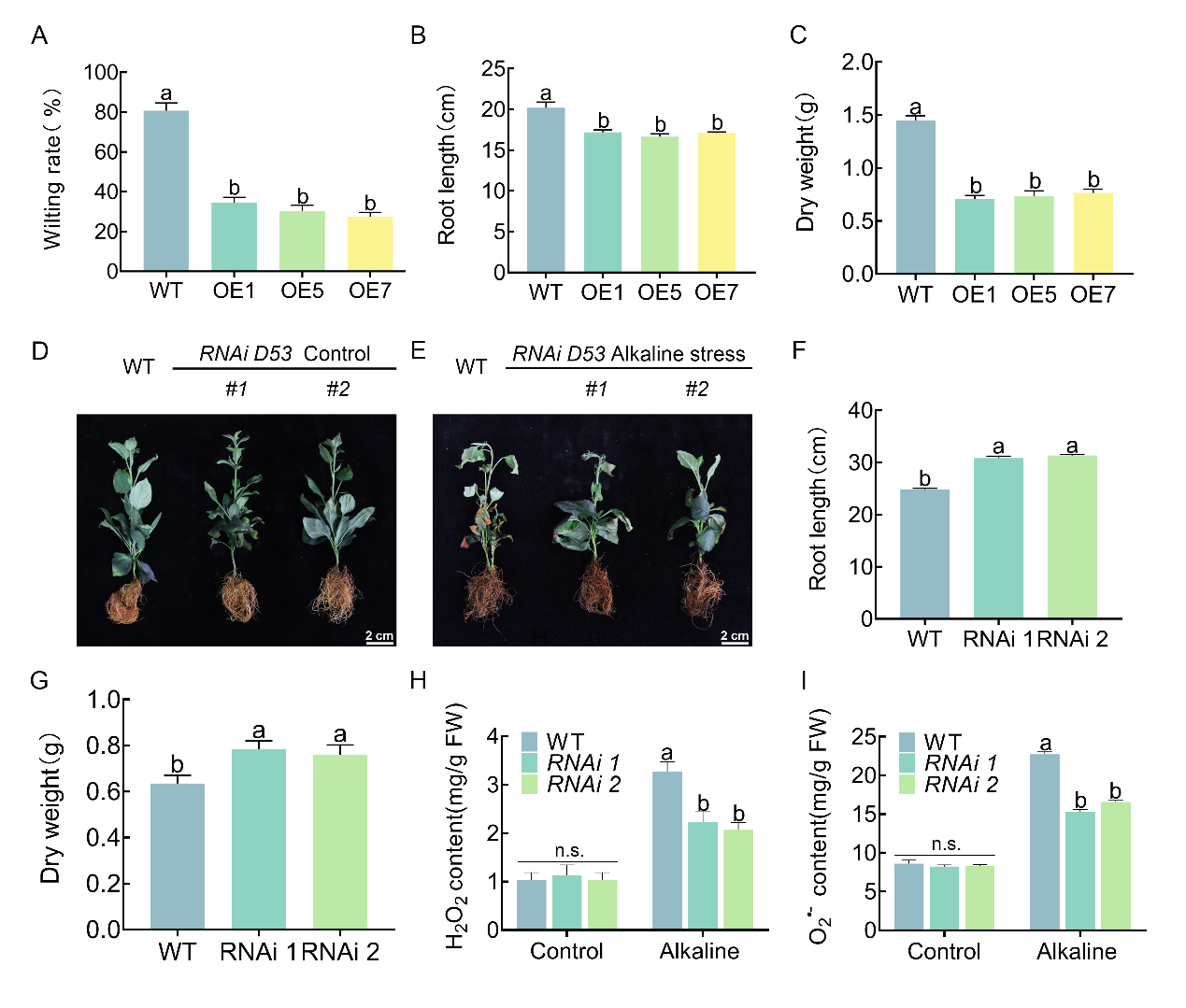


**Figure S5.** Under alkaline salt stress: differences in wilting rate (A), Root length (B), Dry weight (C) between *MdD53-OE* lines and WT; Alkaline salt phenotype of *MdbHLH1-RNAi* lines (D-E); Root length (F); Dry weight (G); and contents of H_2_O_2_ (H) and O_2_·^-^ (I). Data represent means ± SD of three biological replicates. Different lowercase letters indicate significant differences according to Fisher’s LSD test (*P*<0.05). Scale bars: 2 cm (D-E).
