# Supplementary material for: The SL–MdDWARF53–MdbHLH1 module regulates MdAT1-mediated redox homeostasis and alkaline salt tolerance mechanism in apple
Source: Hortic Res. 2026 Mar 11;13(7):uhag089. doi: 10.1093/hr/uhag089 (PMC13271796; doi:10.1093/hr/uhag089)
Supplement: Web_Material_uhag089 [file web_material_uhag089.zip › SUPPORTING TABLE.docx]

**SUPPORTING INFORMATION Table**

The following materials are available in the online version of this article.

**Table S1.** The primers used in this study

| **Primers name** | **Primers sequence (5’→3’)** |
| --- | --- |
| qBHLH1-F1 | CCCGGACATGGACAAGCAAA |
| qBHLH1-R1 | AACTGCACTTCGCCTTCGTA |
| qBHLH1-F2 | GCAACATTTGAGCTTGCCGA |
| qBHLH1-R2 | CCGCATCCTTCTCATCCTCTC |
| qD53-F | GTTCCATTTGGTGGATTCTT |
| qD53-R | ACTGTCATTCTTAGCCTTTG |
| qAT1-F | CGCAGTATCCTGATTTGTATGG |
| qAT1-R | TCAACAGATTTCAGTTCGTCCTC |
| qAT2-F | CTACGAATAGAAAGAAACGCAGGTC |
| qAT2-R | GGTAGCGGACAGCAGGCACAT |
| Actin-F | CTTCAATGTGCCTGCCATGTAT |
| Actin-R | AATTTCCCGTTCAGCAGTAGTG |
| MdD53-cLUC-F | cggggcggtacctccggatccATGCCTACGCCGGTTACTGTA |
| MdD53-cLUC-R | acgacggccagtgccaagcttTCATTTTAGTACAATGCTAGGGAGAAG |
| MdBHLH1-nLUC-F | acgggggacgagctcggtaccATGAGCAGTCTTTTATACAAATACAAT |
| MdBHLH1-nLUC-R | cagtcgacgcgttgtggatccTTTTTGTTTGCTCGAACAACTGC |
| MdD53-AD-F | tgcctctcccgaattATGCCTACGCCGGTTACTGT |
| MdD53-AD-R | tccaaagcttctcgaTTTTAGTACAATGCTAGGGAGAAGATAAGTTT |
| MdBHLH1-BD-F | atggccatggaggccgaattcATGAGCAGTCTTTTATACAAATACAATC |
| MdBHLH1-BD-R | ccgctgcaggtcgacggatccTTTTTGTTTGCTCGAACAACTGC |
| MdBHLH1-AD-F | gccatggaggccagtgaattcATGAGCAGTCTTTTATACAAATACAATC |
| MdBHLH1-AD-R | cagctcgagctcgatggatccTTATTTTTGTTTGCTCGAACAACTG |
| MdAT1-pAbAi-F | aaatgatgaattgaaaagcttAAACGAAGTTAATGATTGCAACTAAAA |
| MdAT1-pAbAi-R | agcacatgcctcgaggtcgacTTCAAATAGTAACCAAAACCCAGTTG |
| MdAT1-pGreen0800-F | ggtatcgataagcttgatatcTTGCAACTAAAATTTACAGAAGTAAATTAAC |
| MdAT1-pGreen0800-R | cgctctagaactagtggatccTTCAAATAGTAACCAAAACCCAGTTG |
| MdBHLH1-SK-F | cgctctagaactagtggatccATGAGCAGTCTTTTATACAAATACAATC |
| MdBHLH1-SK-R | gataagcttgatatcgaattcTTTTTGTTTGCTCGAACAACTGC |
| MdD53-SK-F | cgctctagaactagtggatccATGCCTACGCCGGTTACTGTA |
| 0MdD53-SK-R | gataagcttgatatcgaattcTCATTTTAGTACAATGCTAGGGAGAAG |
| MdD53-PBI121-F | gaccatgattacgccaagcttATGCCTACGCCGGTTACTGTA |
| MdD53-PBI121-R | ggactgaccacccggggatccTCATTTTAGTACAATGCTAGGGAGAAG |
| MdAT1-PBI121-F | gagaacacgggggactctagaATGGATTCTGGTAAGAGCTGGG |
| MdAT1-PBI121-R | ataagggactgaccacccgggAAAACATATACTACAAGGGTTGCAACA |
| MdBHLH1-PRI101-F | ttgatacatatgcccgtcgacATGAGCAGTCTTTTATACAAATACAATCC |
| MdBHLH1-PRI101-R | gcccttgctcaccatggatccTTTTTGTTTGCTCGAACAACTGC |
| MdBHLH1-PBI121-F | gagaacacgggggactctagaATGAGCAGTCTTTTATACAAATACAAT |
| MdBHLH1-PBI121-R | ataagggactgaccacccgggTTTTTGTTTGCTCGAACAACTGC |
| MdBHLH1-pET32a-F | gccatggctgatatcggatccATGAGCAGTCTTTTATACAAATACAATC |
| MdBHLH1-pET32a-R | tgcggccgcaagcttgtcgacTTTTTGTTTGCTCGAACAACTGC |
| MdBHLH1-GST-F | TTCCAGGGGCCCCTGATGAGCAGTCTTTTATACAAATACAATCC |
| MdBHLH1-GST-R | CTCGAGTCGACCCGGTTTTTGTTTGCTCGAACAACTGC |
| MdBHLH1-CYFP-F | gagaacacgggggacgagctcATGAGCAGTCTTTTATACAAATACAAT |
| MdBHLH1-CYFP-R | atcgtatgggtacatgtcgacTTTTTGTTTGCTCGAACAACTGC |
| MdD53-NYFP-F | gagctcggtacccggggatccATGCCTACGCCGGTTACTGTA |
| MdD53-NYFP-R | caacttttgctccatgtcgacTTTTAGTACAATGCTAGGGAGAAGATAA |
| AT1-Bio-F | TTTCCTACGCAGTGGTCACGTGCGGTCTCTGAGGTACTTC |
| AT1-Bio-R | GAAGTACCTCAGAGACCGCACGTGACCACTGCGTAGGAAA |
